# A novel deletion variant in *TRAPPC2* causes spondyloepiphyseal dysplasia tarda in a five-generation Chinese family

**DOI:** 10.1186/s12881-020-01052-8

**Published:** 2020-05-29

**Authors:** Cai Zhang, Caiqi Du, Juan Ye, Feng Ye, Renfa Wang, Xiaoping Luo, Yan Liang

**Affiliations:** 1 Department of Pediatrics, Tongji Hospital, Tongji Medical College, Huazhong University of Science and Technology, Wuhan, China; 2 Department of Radiology, Tongji Hospital, Tongji Medical College, Huazhong University of Science and Technology, Wuhan, China

**Keywords:** Spondyloepiphyseal dysplasia tarda, Short stature, TRAPPC2, SEDL

## Abstract

**Background:**

Spondyloepiphyseal dysplasia tarda (SEDT) is a rare X-linked recessive inherited osteochondrodysplasia caused by mutations in the *TRAPPC2* gene. It is clinically characterized by disproportionate short stature and early onset of degenerative osteoarthritis. Clinical diagnosis can be challenging due to the late-onset of the disease and lack of systemic metabolic abnomalites. Genetic diagnosis is critical in both early diagnosis and management of the disease. Here we reported a five-generation Chinese SEDT family and described the novel molecular findings.

**Methods:**

Detailed family history and clinical data were collected. Genomic DNA was extracted from venous blood samples of family members. The exons of genes known to be associated with skeletal disorders were captured and deep sequenced. Variants were annotated by ANNOVAR and associated with multiple databases. Putative variants were confirmed by Sanger sequencing. The identified variant was classified according to the American College of Medical Genetics (ACMG) criteria.

**Results:**

The proband was a 27-year-old Chinese male who presented with short-trunk short stature and joint pain. His radiographs showed platyspondyly with posterior humping, narrow hip-joint surfaces, and pelvic osteosclerosis. A pedigree analysis of 5 generations with 6 affected males revealed an X-linked recessive mode of inheritance. Affected males were diagnosed as SEDT according to the clinical and radiological features. Next-generation sequencing identified a novel variant of c.216_217del in the exon 4 of *TRAPPC2* gene in the proband and other affected males. This variant resulted in the shift of reading frame and early termination of protein translation (p.S73Gfs*15). The mother and maternal female relatives of the proband were heterozygous carriers of the same variant, while no variations were detected in this gene of his father and other unaffected males. Based on the ACMG criteria, the novel c.216_217del variant of the *TRAPPC2* gene was the pathogenic variant of this SEDT family.

**Conclusion:**

In this study we identified the novel pathogenic variant of of c.216_217del in the gene of *TRAPPC2* in this five-generation Chinese SEDT family. Our findings expand the clinical and molecular spectrum of SEDT and helps the genetic diagnosis of SEDT patients.

## Background

Spondyloepiphyseal dysplasia tarda (SEDT, OMIM 313400), is a rare inherited, late-onset osteochondrodysplasia, characterized by disproportionately short stature and premature osteoarthritis [1]. The estimated prevalence in Britain is about 1.7 per 1,000,000 [2]. It is an X-linked recessive inherited disease whereby only males are affected. The patients have normal growth at birth, but manifestions of this condition become evident after the age of 5–10 years old, with growth retardation, joint pain, and limited mobility [1]. The causative gene of SEDT is *TRAPPC2* (previously named *SEDL*) located on Xp22, which encodes a protein of 140 amino acids, traffic protein particle complex subunit 2 (TRAPPC2), also known as Sedlin [3]. To date, about 50 *TRAPPC2* variants responsible for SEDT have been reported (Human Gene Mutation Database, HGMD; http://www.hgmd.cf.ac.uk/ac), and the most common type was deletion mutation. Here, we report a Chinese familial case of SEDT that harbors a novel deletion mutation in *TRAPPC2*.

## Methods

### Genetic analysis

**Fig. 1:**
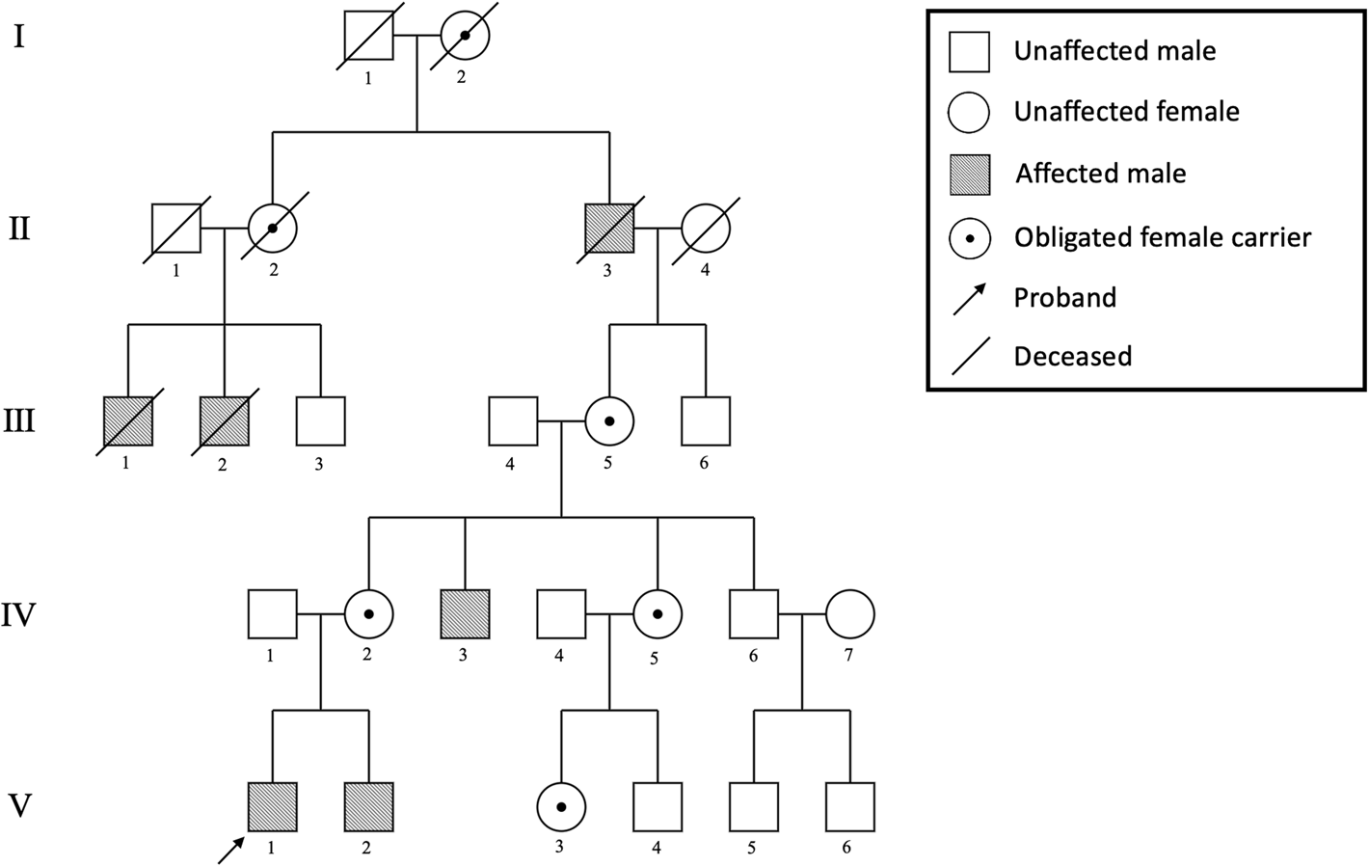
Family pedigree of the Chinese family with short stature. Individuals include: males (squares), females (circles), deceased individuals (symbols with a crossing line), unaffected individuals (open symbol), affected individuals (slashed symbol), and X-linked carriers (denoted with a dot in the middle of symbol)

With the consent of the participants, venous blood samples from 11 members of the family (III4, III5, IV1, IV2, IV3, IV5, IV6, V1, V2, V3, V4 in Fig. 1), and genomic DNA was extracted from each sample. The DNA librariy was prepared by DNA sample prep reagent set (MyGenostics, Beijing). 219 exons known to be associated with skeletal disorders were captured using GenCap WES capture kit (MyGenostics, Beijing) and deep sequenced on the Illumina HiSeq X ten platform (Illumina, California) [4]. Variants were identified by GATK and annotated with ANNOVAR, and were further associated with multiple databases, such as,1000 Genomes, ESP6500, dbSNP, EXAC, Inhouse (MyGenostics), HGMD, and predicted by SIFT, PolyPhen-2, MutationTaster, GERP++ [5]. Sanger sequencing was performed to confirm the potentially pathogenic variants. The identified variant was classified according to the American College of Medical Genetics (ACMG) criteria.

## Results

### Clinical report

A 27-year-old Chinese male of short stature (the proband, case V1 in Fig. 1) was presented for genetic counseling due to multiple members of short stature in the family. The pedigree of the Chinese family with short stature is shown in Fig. 1.

**Fig. 2:**
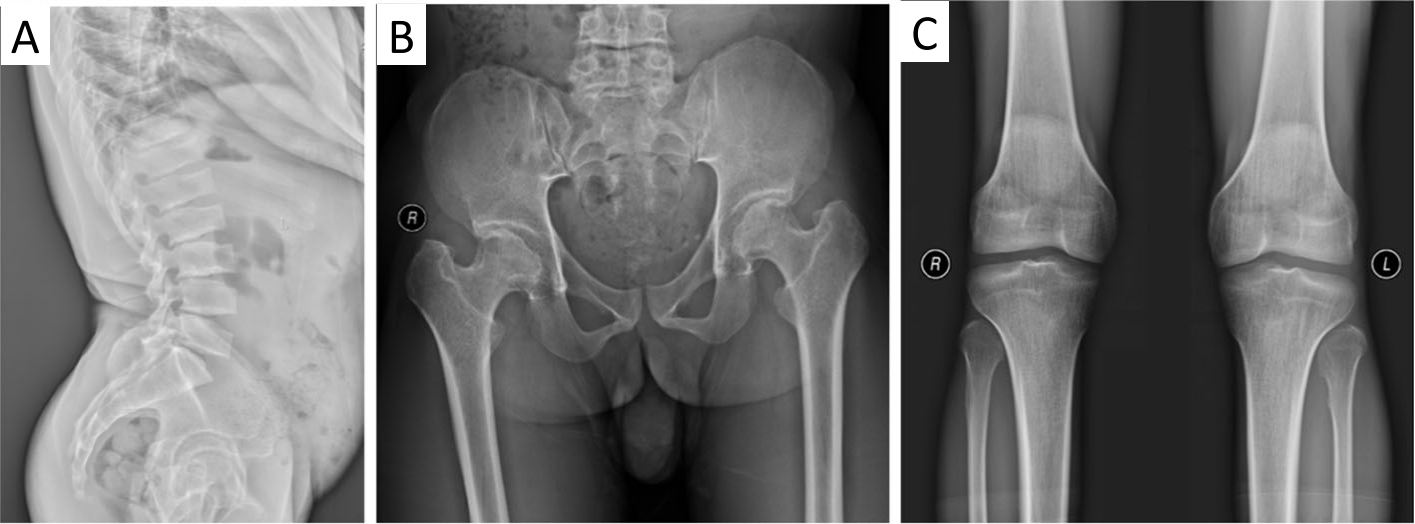
X-ray radiographs of the proband. **a**, the radiograph of the lateral lumbosacral spine showed platyspondyly and dysplasia with posterior humping. **b**, the radiograph of the hips showed flattened femoral heads, narrow hip-joint surfaces, and osteosclerosis of the pelvis. **c**, the radiograph of the knees displayed flattened tibial plateaus and irregular surface of lateral femur condyles

The proband (V1) showed stunted growth after the age of 10 years, and experienced mild back pain provoked by exercise after the age of 26. He was 137.3 cm (− 5.87SDS) tall, with an arm span of 143.5 cm and an upper to lower body segment ratio of 0.91. His chest was barrel-shaped, with no notable abnormalities in vertebrae or limbs. The lateral radiograph of the lumbosacral spine showed platyspondyly and dysplasia with posterior humping (Fig. 2a). Flattened femoral heads, narrow hip-joint space and osteosclerosis were observed in the radiograph of the pelvis (Fig. 2b). The radiograph of the knees showed flattened tibial plateaus and irregular surfaces of lateral femoral condyles (Fig. 2c). He was born to non-consanguineous and normal parents (IV1 and IV2). His father’s and mother’s height were 170 cm and 153 cm, respectively. There were 2 other living affected males in the family, identified as the younger bother (V2) and the maternal uncle (IV3) of the proband, who had similar symptoms and manifestations. They all showed stunted growth around the age of 10–12 years. Case V2, who was 26 years old and 136 cm tall, complained of mild back and hip pain provoked by exercise. He had a barrel-shaped chest and no abnomalites of the vertebrae or limbs. Case IV, who was 50 years old and 134 cm tall, complained of severe back, hip and knee pain, with notable scoliosis and limitation of motion after the fourth decade of his life.

The heights of the affected cases were from 134 to 137 cm, and their chests were all barrel-shaped. X-ray radiography showed typical characteristics of SEDT, including platyspondyly, dysplasia with posterior humping of the vertebral bodies, and osteoarthritic changes in the hip, joints, and the knees. The affected cases were all male with the average height of 136.2 cm, while the heights of the women in this family were normal (the average height was 155.3 cm). The proband as well as the maternal uncle and younger bother of the proband were affected. However, the height of the proband’s father was normal. These findings indicated an X-linked recessive mode of inheritance. SEDT was diagnosed based on the clinical manifestations, the radiological features, and the inheritance pattern of the family.

### Genetic analysis

**Fig. 3:**
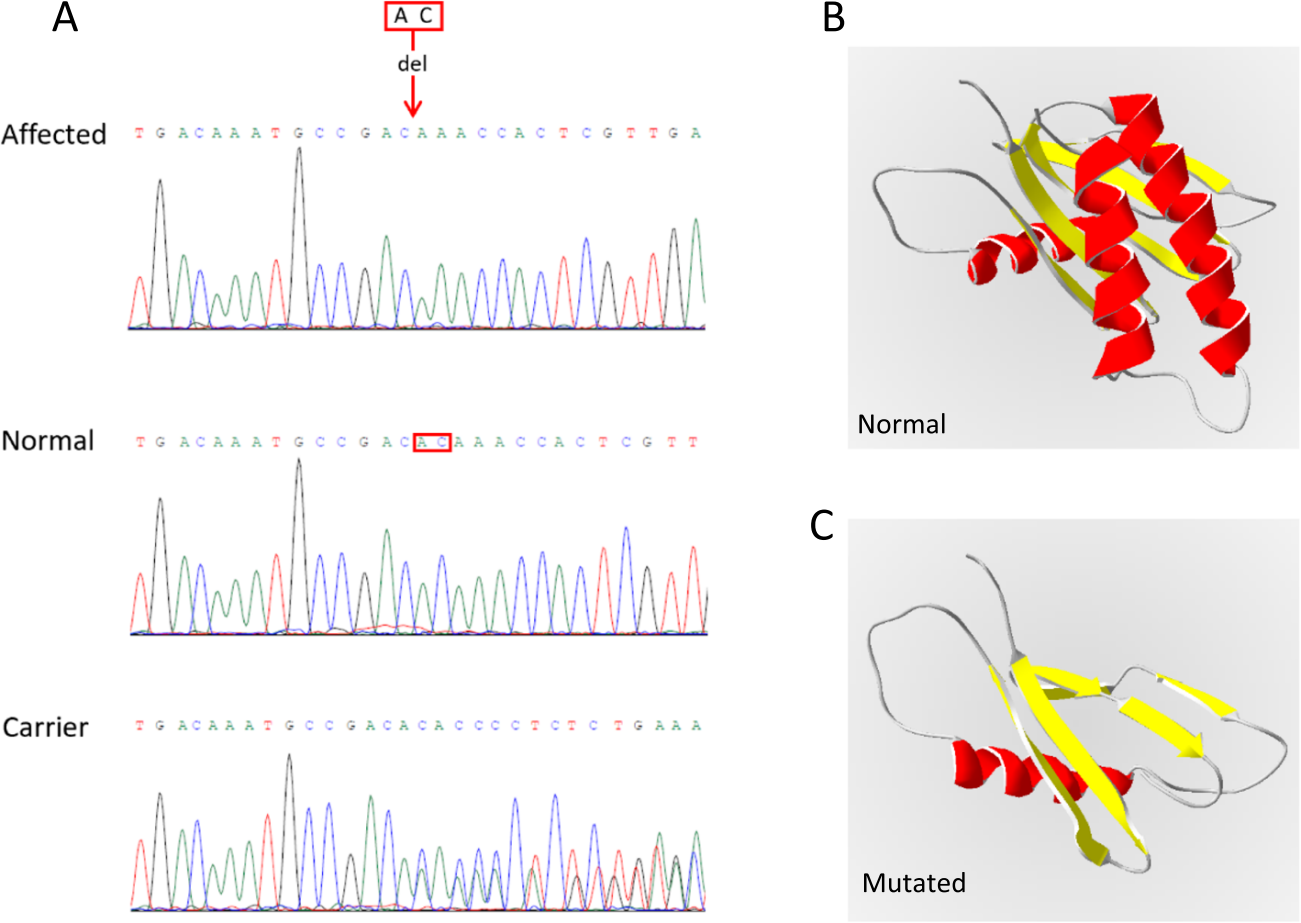
Sequence analysis of the family and the predicted structure of the mutated protein. **a**, Sequencing chromatograms of affected members (V1, V2, IV3), unaffected members (IV1, III4, IV6, V4), and carriers (IV2, III5, IV5, V3) from this family. The red arrow indicates the locus of the c.216_217del variant, and the red box indicates the corresponding normal sequence. **b**, the predicted secondary structure of the normal TRAPPC2 protein. **c**, the predicted secondary structure of the mutated TRAPPC2 protein (p. S73Gfs*15)

Genomic DNA sequencing of the proband revealed a novel hemizygous variant of c.216_217del in the exon 4 of the *TRAPPC2* gene (NM_001011658.4) (Fig. 3a), which resulted in the frameshifts and early termination of protein translation (p.S73Gfs*15) (Fig. 3b and Fig. 3c). There was no variant of *TRAPPC2* detected in the father of the proband (IV1) (Fig. 3a), while the mother (IV2) was a heterozygous carrier of the c.216_217del variant (Fig. 3a). This variant appeared to be novel, which is not included in the databased mentioned above, and has not been reported in normal populations. Further analysis of this family showed that the same variant was found in affected case IV3 and V2, and all maternal female relatives of the proband (III5, IV5, V3) were carriers of the variant. However, no *TRAPPC2* variant was detected in phenotypic normal male members (III4, IV6, V4), suggesting the segregation of the variant with the phenotype. Based on the ACMG criteria, the novel c.216_217del variant of the *TRAPPC2* gene was the pathogenic variant of this SEDT family.

## Discussion

SEDT is a rare X-linked recessive, progressive osteochondrodysplasia involving vertebral bodies and weight-bearing joints [1]. The late-onset of the disease and lack of systemic metabolic abnomalites result in difficulties in diagnosis. Thus genetic testing is critical in both early and prenatal diagnosis.

The causative gene *TRAPPC2* is located on Xp22, which spans a genomic region of approximately 20 kb and contains six exons. The coding region encompassed by exons 3–6 is 420 bp in size and encodes 140-amino acid protein TRAPPC2 [3]. TRAPPC2 binds to other components of traffic protein particle complex (TRAPP) and serves as an adaptor for the formation of the complex, playing a critical role in the traffic of vesicles between the endoplasmic reticulum (ER) and the Golgi complex as well as being involved in the regulation of the ER export of procollagen [6, 7]. Moreover, it was also reported that TRAPPC2 interacts with multiple transcription factors and perhaps modulates the expression of genes involved in skeletal development [8].

Most of the variants of the *TRAPPC2* gene cause premature termination of translation, leading to degradation of partially translated peptides; while some variants result in misfolding of the mutant protein, invoking the protein degradation pathway [9]. All of them deprive the cells of TRAPPC2 function, leading to osteochondrodysplasia. In this study, c.216_217del variant of the *TRAPPC2* gene resulted in the frameshifts and early termination of protein translation (p.S73Gfs*15), leading to degradation of truncated protein and loss of the TRAPPC2 protein function (Fig. 3b and Fig. 3c).

**Fig. 4:**
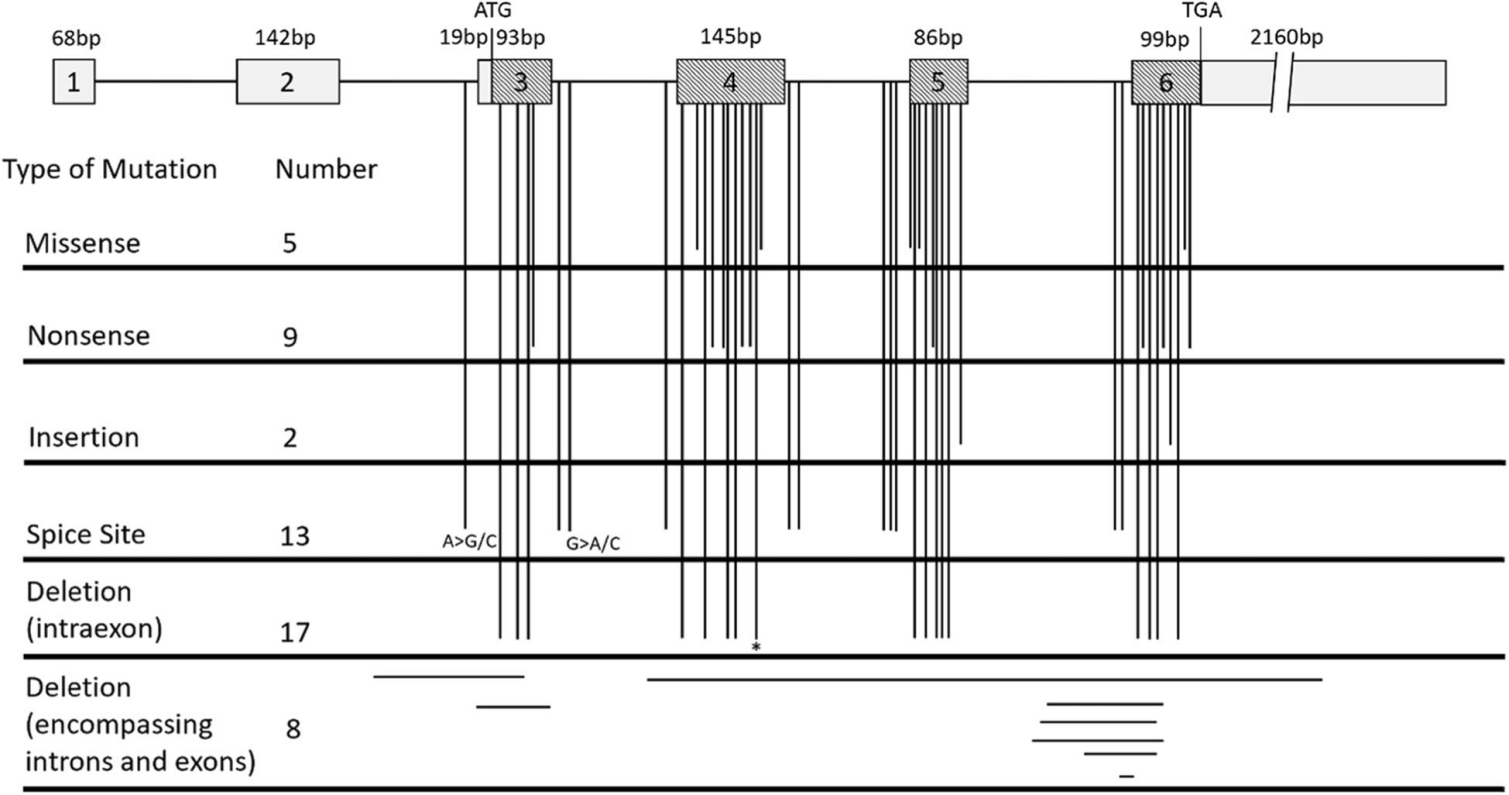
The summary of identified variants in the *TRAPPC2* gene illustrated by a schematic diagram. The human *TRAPPC2* gene consists of six exons that span approximately 20 kb of genomic DNA. The 420 bp coding region (hatched boxes) is organized into four exons (exon 3 to exon 6). Non-coding exons (open boxes) consist of exons 1 and 2, the 5′ portion of exon 3, and the 3′ portion of exon 6. The translation Start (ATG) and Stop (TGA) codons in exon 3 and 6, respectively, are indicated. Introns are indicated by a line (not to scale). The locations of the 54 variants are indicated, including the variant identified in this study (denoted by the asterisk)

According to HGMD, there are 54 different pathogenic variants that have been reported so far including the present study, and a majority of them originated from Europe, China, Japan, and Austrialia (Table 1). The variants occur most frequently in exon 4–6 (Fig. 4), which are important regions of protein binding and maintaining the three-dimensional structure of TRAPPC2 protein [9, 37]. Among these variants, there are 25 deletion mutations (46.3%), 13 splicing site mutations (24%), 9 nonsense mutations (16.7%), 5 missense mutations (9.3%), and 2 insertion mutations (3.7%) (Fig. 4). The frequency of the deletion mutations is unusually high, particularly for a gene encoding only 140 amino acids. Homologous recombination and slipped mispairing of five pseudogenes of *TRAPPC2* (*SEDLP3-SEDLP7*) on chromosome Yq11.23 may provide a possible explanation of the high frequency of deletion mutations [29]. The novel variant found in this study is a deletion mutation located on exon 4, leading to frameshifts and premature termination of protein translation. There are 3 reported pathogenic variants which are adjacent to it. Among them, two are nonsense mutations that results in truncated protein, while one is a missense mutation that results in misfolding and degradation of the mutated protein [11, 22, 23]. These causative variants suggest that the 5′ region of exon 4 is critical in the structure and function of TRAPPC2.

**Table 1:** Summary of identified pathogenic variants in the *TRAPPC2* gene

Number	Gene Region	Nucleotide Change	Mutation Type	Predicted Amino Acid Change	Origin	Year of Report
1	Intron 2	c.-19-2A > C	Spice Site	–	Chinese	2003 [10]
2	Intron 2	c.-19-2A > G	Spice Site	–	French	2001 [11]
3	Intron 2 and Exon 3	deletion 1763 bp across I2E3 boundary	Deletion	–	Japanese	2001 [12]
4	Exon 3	c.6delT	Deletion	p.S4Afs*4	Brazilian	2018 [13]
5	Exon 3	c.40delG	Deletion	p.D14Ifs*23	Korean	2012 [14]
6	Exon 3	c.53_54delTT	Deletion	p.F18*	Australian	1999 [3]
7	Exon 3	c.61G > T	Nonsense	p.E21*	Chinese	2014 [15]
8	Exon 3	Exon3del	Deletion	–	Australian	2001 [11]
9	Intron 3	c.93 + 1G > A	Spice Site	–	Japanese	2014 [16]
10	Intron 3	c.93 + 5G > C	Spice Site	–	Chinese	2013 [17]
11	Intron 3	c.93 + 5G > A	Spice Site	–	Chinese	2015 [18]
12	Intron 3	94-2A > G	Spice Site	–	Japanese	2018 [19]
13	Exon 4–6	deletion Exon 4–6	Deletion	–	European	2004 [20]
14	Exon 4	c.100delC	Deletion	p.H34Ifs*3	European	2004 [20]
15	Exon 4	c.139G > T	Missense	p.D47Y	Danish	2001 [11]
16	Exon 4	c.157_158delAT	Deletion	p.M53Vfs*34	Australian	1999 [3]
17	Exon 4	c.167C > A	Nonsense	p.S56*	Germany	2003 [21]
18	Exon 4	c.182 T > A	Nonsense	p.L61*	Mexican	2001 [11]
19	Exon 4	c.183_184delGA	Deletion	p.K62Nfs*25	European	2004 [20]
20	Exon 4	c.191_192delTG	Deletion	p.V64Gfs*23	Australian	1999 [3]
21	Exon 4	c.209G > A	Nonsense	p.W70*	Chinese	2008 [22]
22	Exon 4	c.210G > A	Nonsense	p.W70*	European	2001 [23]
23	Exon 4	c.216_217del	Deletion	p.S73Gfs*15	Chinese	Present Study
24	Exon 4	c.218C > T	Missense	p.S73L	Norwegian	2001 [11]
25	Intron 4	c.238 + 1A > G	Spice Site	–	Chinese	2009 [24]
26	Intron 4	c.238 + 4 T > C	Spice Site	–	Italian	2003 [25]
27	Intron 4	c.239-11_239-9delAAT	Spice Site	–	Turkish	2014 [26]
28	Intron 4	c.239-11_239-4delAATTATTT	Spice Site	–	German	2001 [11]
29	Intron 4	c.239-10_239-7delATTA	Spice Site	–	North American	2001 [11]
30	Exon5	c.239A > G	Missense	p.H80R	Chinese	2008 [27]
31	Exon 5	c.241_242delAT	Deletion	p.M81Efs*6	French	2001 [11]
32	Exon 5	c.248 T > C	Missense	p.F83S	British	2001 [28]
33	Exon 5	c.262_266delGACAT	Deletion	p.D88Kfs*11	Canadian	2001 [11]
34	Exon 5	c.271C > T	Nonsense	p.Q91*	–	2000 [29]
35	Exon 5	c.271_275delCAAGA	Deletion	p.Q91Rfs*8	North American	2000 [30]
36	Exon 5	c.272_273delAA	Deletion	p.Q91Rfs*9	Finnish	2001 [11]
37	Exon 5	c.293delT	Deletion	p.F98Sfs*10	Chinese	2003 [31]
38	Exon 5	c.320dupT	Insertion	p.F109Vfs*7	Native Australian	2001 [11]
39	Intron 5	c.325-10_325-4delTCTTTCCinsAA	Spice Site	–	French	2001 [11]
40	Intron 5	c.325-2A > C	Spice Site	–	Australian	2001 [11]
41	Intron 5 and Exon 6	deletion 1330 bp across I5E6	Deletion	–	Australian	2001 [11]
42	Intron 5 and Exon 6	deletion 1335 bp across I5E6 boundary	Deletion	–	Belgian	2003 [25]
43	Intron 5 and Exon 6	deletion 1371–1445 bp across I5E6 boundary	Deletion	–	European	2001 [23]
44	Intron 5 and Exon 6	deletion 750 bp across I5E6 boundary	Deletion	–	European	2001 [23]
45	Intron 5 and Exon 6	c.325-2_335delAGTTTTCAATGAA	Deletion	p.F109Sfs*3	Chinese	2004 [32]
46	Exon 6	c.328delT	Deletion	p.S110Qfs*1	European	2004 [20]
47	Exon 6	c.329C > A	Nonsense	p.S110*	Chinese	2002 [33]
48	Exon 6	c.333_336delGAAT	Deletion	p.M111Ifs*29	Slovakian	2003 [25]
49	Exon 6	c.345_346delTG	Deletion	p.Y115*	European	2004 [20]
50	Exon 6	c.364C > T	Nonsense	p.R122*	European	2001 [23]
51	Exon 6	c.370dupA	Insertion	p.S124Kfs*3	Chinese	2009 [34]
52	Exon 6	c.387delA	Deletion	p.V130Ffs*9	Jewish Ashkenazi	2004 [35]
53	Exon 6	c.389 T > A	Missense	p.V130D	Japanese	2001 [11]
54	Exon 6	c.391C > T	Nonsense	p.Q131*	Japanese	2002 [36]

The correlation of genotype and SEDT phenotype is still unknown. In this study, cases V1 (27 years) and V2 (26 years), complained of only mild joint pain without skeletal abnomalities. But case IV3, who was age 50 years, showed severe joint pain, scoliosis, and limited movement. These intrafamilial phenotypic differences could have possibly resulted from the penetrance of *TRAPPC2* mutations and the course of progressive osteoarthritis. Gedeon et al. studied 30 cases of SEDT patients and found no obvious genotype/ phenotype correlation, but a suggestion that variants close to the 5′ end of the *TRAPPC2* gene resulted in severe clincial presentations compared with variants close to the 3′ end. Patients with variants affecting exons 5 and 6 showed a milder condition, with little or no hip pain and neither kyphosis nor scoliosis. Variants in exons 3 and 4 result in kyphosis and scoliosis, severe pain evident earlier in life and a more debilitating set of complications [11].

## Conclusions

In conclusion, we report a novel pathogenic variant (c.216_217del) of the *TRAPPC2* gene in this SEDT family. Our findings enable carrier detection, genetic counseling, and asymptomatic/prenatal diagnosis. Identification of the novel disease-causing mutaiton will assist in further elucidation of the role of the TRAPPC2 protein in bone growth.

## Data Availability

The data generated during the current study are available on online public repository ClinVar (https://submit.ncbi.nlm.nih.gov/clinvar/). An accession number (VCV000694600.1) for the novel variant identified in this study has also been allocated (https://www.ncbi.nlm.nih.gov/clinvar/variation/ 694600/). The raw datasets generated and analysed during the current study are not publicly available in order to protect participant confidentiality.

## Abbreviations

SEDT: Spondyloepiphyseal dysplasia tarda
TRAPPC2: Traffic protein particle complex subunit 2
HGMD: Human Gene Mutation Database
